# Botanical ethnoveterinary therapies in three districts of the Lesser Himalayas of Pakistan

**DOI:** 10.1186/1746-4269-9-84

**Published:** 2013-12-20

**Authors:** Arshad Mehmood Abbasi, Shujaul Mulk Khan, Mushtaq Ahmad, Mir Ajab Khan, Cassandra Leah Quave, Andrea Pieroni

**Affiliations:** 1 Department of Environmental Sciences, COMSATS Institute of Information Technology, Abbottabad 22060, Pakistan; 2 Department of Botany, Hazara University Mansehra, Mansehra 21300, Pakistan; 3 Department of Plant Sciences, Quaid-i-Azam University, Islamabad 45320, Pakistan; 4 Department of Dermatology, Emory University School of Medicine, 1518 Clifton Rd NE, CNR Bldg. 5000, Atlanta, GA 30322, USA; 5 Center for the Study of Human Health, Emory College of Arts and Sciences, 550 Asbury Circle, Candler Library 107, Atlanta, GA 30322, USA; 6 University of Gastronomic Sciences, Piazza Vittorio Emanuele 9, Pollenzo I-12042 Bra/Pollenzo, Italy

**Keywords:** Medicinal plants, Ethnobotany, Ethnoveterinary, Lesser Himalayas, Pakistan

## Abstract

**Background:**

Ethnoveterinary knowledge is highly significant for persistence of traditional community-based approaches to veterinary care. This is of particular importance in the context of developing and emerging countries, where animal health (that of livestock, especially) is crucial to local economies and food security. The current survey documents the traditional veterinary uses of medicinal plants in the Lesser Himalayas-Pakistan.

**Methods:**

Data were collected through interviews, focus groups, participant observation, and by administering questionnaires. A total of 105 informants aged between 20–75 years old who were familiar with livestock health issues (i.e. farmers, shepherds, housewives and herbalists) participated in the study.

**Results:**

A total of 89 botanical taxa, belonging to 46 families, were reported to have ethnoveterinary applications. The most quoted families were Poaceae (6 taxa), Fabaceae (6), Asteraceae (5), and Polygonaceae (5). *Adhatoda vasica* was the most cited species (43%), followed by *Trachyspermum ammi* (37%), and *Zanthoxylum armatum* var. *armatum* (36%). About 126 medications were recorded against more than 50 veterinary conditions grouped into seven categories. The highest cultural index values were recorded for *Trachyspermum ammi, Curcuma longa, Melia azedarach*, *Zanthoxylum armatum* var. *armatum* and *Adhatoda vasica.* The highest informant consensus factor was found for pathologies related to respiratory and reproductive disorders. Comparison with the local plant-based remedies used in human folk medicine revealed that many of remedies were used in similar ways in local human phytotherapy. Comparison with other field surveys conducted in surrounding areas demonstrated that approximately one-half of the recorded plants uses are novel to the ethnoveterinary literature of the Himalayas.

**Conclusion:**

The current survey shows a remarkable resilience of ethnoveterinary botanical knowledge in the study area. Most of the species reported for ethnoveterinary applications are wild and under threat. Thus, not only is it imperative to conserve traditional local knowledge of folk veterinary therapies for bio-cultural conservation motives, but also to assist with *in-situ* and *ex-situ* environmental conservation initiatives, which are urgently needed. Future studies that focus on the validation of efficacy of these ethnoveterinary remedies can help to substantiate emic concepts regarding the management of animal health care and for rural development programs.

## Introduction

Ethnoveterinary medicine is a broad field encompassing people’s beliefs, skills, knowledge and practices related to veterinary health care [1]. Medicinal plants traditionally used in the treatment of animal diseases play a crucial role in local health modalities. Specifically, phytotherapeutics often represent the primary form of therapy in rural veterinary care as allopathic modalities remain inaccessible, especially in the developing world [2]. Therefore, local knowledge of ecological resources for veterinary care is of particular importance to pastoral and agro-pastoral communities that rely heavily on livestock for their livelihood and food security. However, traditional ethnoveterinary knowledge is still mainly orally transmitted from generation to generation (i.e., in the form of traditional remedies, poems, drawings stories, folk myths, proverbs and songs). Due to the nature of oral transmission, this form of local knowledge remains fragile and threatened, and presents an urgent need for being recorded and documented.

An increasing number of studies have very recently focused on the documentation of local ethnoveterinary practices in South Asia [3-24]. These studies hold potential for having a tremendous impact on the Himalayan region, in particular, where efforts for sustaining endogenous development and ultimately improving the health and well-being of both animals and humans is still largely neglected. Pakistan has a very large livestock population composed of a number of local breeds that are well adapted to local conditions. In particular, there are an estimated 27 million buffaloes, 30 million cattle, 27 million sheep, 54 million goats, one million camels, 0.3 million horses, 4 million asses, 0.2 million mules and 74 million poultry in Pakistan [25].

The objectives of this field study were multifold: 1. to record the local knowledge related to medicinal plants used for treating animal diseases in the Lesser Himalayan region in Pakistan; 2. to compare the collected data with the traditional medical knowledge devoted to humans in the same region; 3. to compare the collected data with those of other ethnoveterinary studies conducted in the Himalayan region over the last decades; 4. to assess their cultural importance and the consensus among the informants regarding cited veterinary pathologies; and 5. to examine local perceptions of factors that threaten wild medicinal plant resources.

## Materials and methods

### Study site

**Figure 1:**
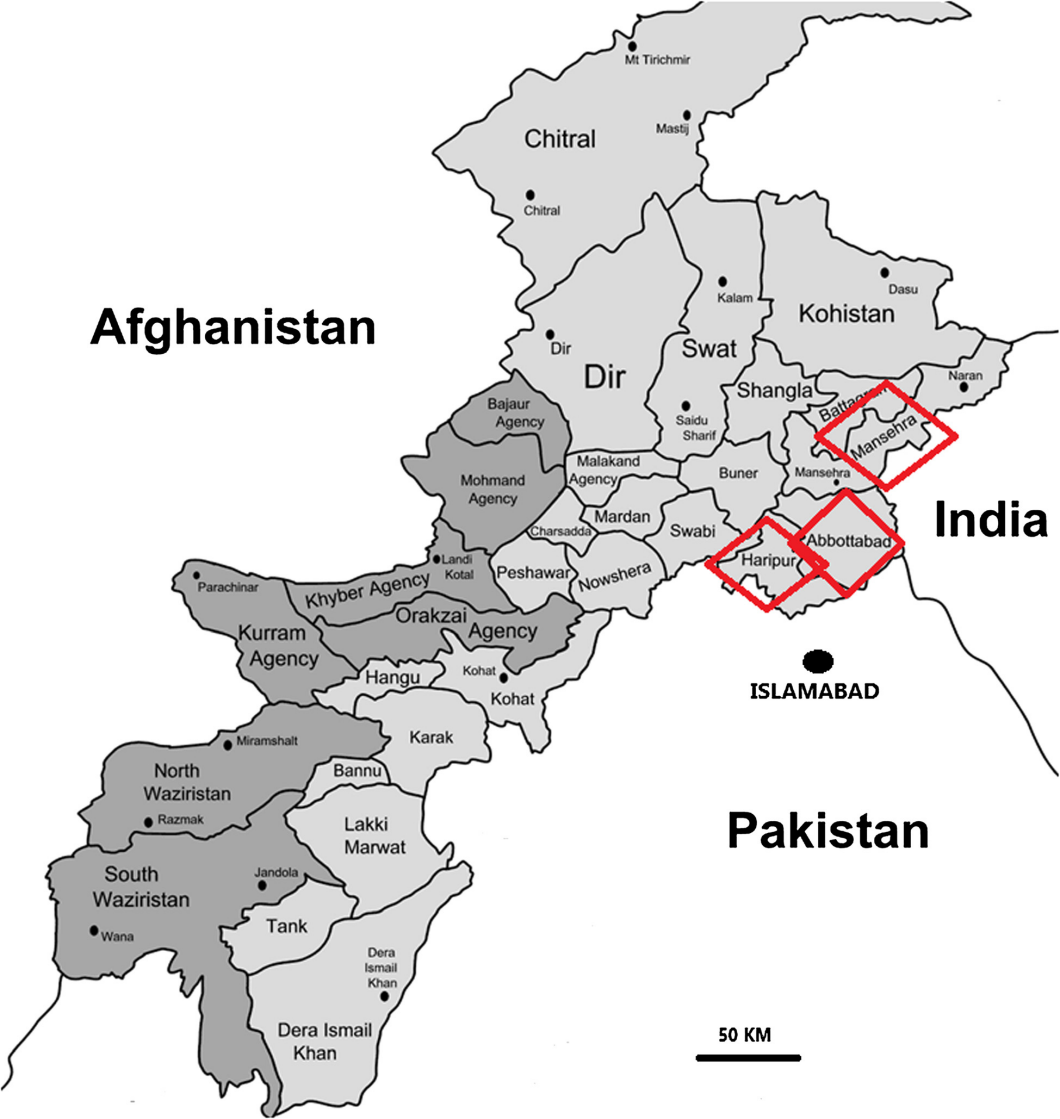
Map of the study area.

An ethnobotanical study was conducted from March 2010 to April 2013 in different locations of the Lesser Himalayas, which is a hotspot for plant biodiversity in Pakistan. Fifty-five localities in three districts (Haripur, Abbottabad and Mansehra) within the Khyber Pakhtunkhwa (KPK) province were selected for inclusion in the study (Figure 1). The Lesser Himalayan range in Pakistan lies between 33°-44′ and 35°-35′ north latitude and between 72°-33′ and 74°-05′ east longitude, comprising an area of 23,295 km^2^. The climate of the area is subtropical in the lowland plains and foot-hills zone and subtropical-sub alpine in middle Himalayas, Siwalik, Murree and entire Hazara hills. The average rainfall varies from 70–90 mm in southern and 100–130 mm in the northern parts. The vegetation of the Lesser Himalayas falls within the subtropical, temperate, sub-alpine and alpine zones. The region is divided into six vegetation zones, namely: the subtropical sub-humid zone, the subtropical humid zone, the temperate humid zone, the sub alpine zone and the zone of the glaciers/snowfields. This area is populated by several ethnic groups (Syed, Abbasi, Karaal, Jadoon, Tanoli, Ghakar, Gujar, and Awan), all speaking the Hindko dialect of the Western Punjabi, and belonging in turn to the Indo-Aryan (Indic) language family spoken in Northern Pakistan.

### Ethnobotanical data collection

Ethnobotanical surveys were conducted in all four seasons. Participatory rural appraisal (PRA) approaches were adopted during fieldwork and prior informed consent was obtained before conducting interviews. Information regarding ethnoveterinary practices was collected through semi-structured interviews and guided fieldtrips with the help of traditional healers. A total of 105 informants (75 males and 30 females), ranging from 20–75 years old and including farmers, shepherds, housewives and herbalists familiar with livestock problems and use of conventional recipes, were interviewed and their responses recorded in detail.

Information regarding the vernacular plant names, part(s) used, methods of preparation, mode/route of application and treated diseases were documented during each interview. Taxonomic identification of the collected plant samples was carried out with the help of Flora of Pakistan[26], The Plant List [27] and by one of the authors (MAK, plant taxonomist). Family nomenclature follows the Angiosperm Phylogeny Group III designations [28]. Additionally, 15 key informants were selected at four locations within three study districts (Haripur, Abbottabad and Mansehra) and specific information regarding the perceived threats for the local medicinal flora was obtained. Following identification of 5 key perceived threats (agricultural land expansion, overharvesting, overgrazing, fuel, fire), we employed pair-wise ranking techniques in which respondents were presented with two threats and chose one from the two threats at a time [29]. Respondent scores were then summed up and ranks for each threat determined by region.

### Data analysis

Cultural importance index (CI) values for each species and mean cultural importance values for each family (mCIf) were calculated as described in a previous quantitative ethnobotanical work [30]. Briefly, CI values of species were calculated based on previously described methods [31] and express the sum of the proportion of informants that mention each species used. The CI values for each species were calculated using the following formula, with UR_i_: use reports in each use-category and N: total number of survey participants:

$$\mathrm{CI}=\sum \frac{UR_{i}}{N}$$

Moreover, we calculated the mean cultural importance (mCI) index of plant species as measured in three study districts (Haripur, Abbottabad, Mansehra) within the Khyber Pkahtunkhaw Province of Pakistan, on the basis of their cultural importance index (CI) calculated for each single district. To calculate the mCIf, CI values of all reported species within a family were added. Regression analysis was performed upon comparison of mCIf with the number of species in each respective family.

Informant consensus on the reported cures for a given group of aliments was calculated as an informant consensus factor (ICF) [32]. All of the quoted veterinary diseases were grouped into seven categories, which included: gastrointestinal disorders, skin infections, parasites/worms, fever/cold/respiratory diseases, reproductive disorders, musculoskeletal disorders and galactagogue remedies. As previously reported [32], we used the following formula, with *n*_*ur*_: number of use citations in each category and *n*_*t*_: number of species used:

$$\mathrm{ICF}=\frac{n_{\mathrm{ur}}-n_{t}}{n_{\mathrm{ur}}-1}$$

Lastly, collected data were compared with previously conducted ethnoveterinary studies carried out in surrounding areas [8,33-45].

## Result and discussion

### Taxonomic diversity of the species

**Figure 2:**
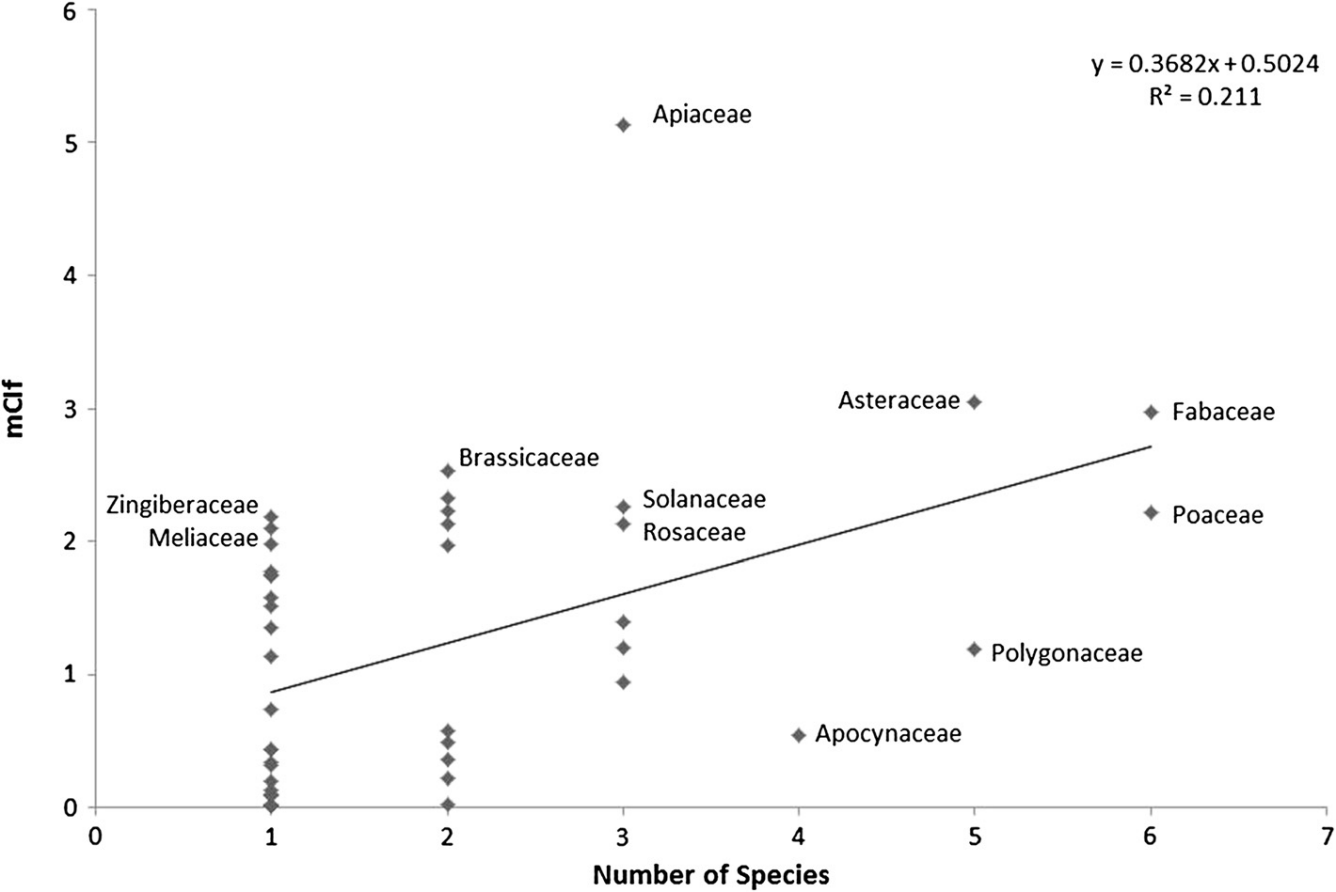
Regression of the cultural importance of the families (mCIf) on the number of species in the family.

A total of 89 plant species belonging 81 genera and 46 families were reported by the study participants against veterinary aliments have been gathered and documented alphabetically along with their local names, parts used, preparations, applications, indications and citation numbers (Table 1). Among the most utilized botanical families, Poaceae and Fabacaeae were ranked first with highest number of species (6 taxa), followed by Asteraceae (5), Polygonaceae (5), and Apocynaceae (4) (Figure 2).

**Table 1:** Botanical ethnoveterinary therapies for treating livestock in communities of the Lesser Himalayas in Pakistan

**Botanical name and voucher number**	**Local name**	**PU**^**a**^	**Preparation and application**	**Veterinary condition**	**AT**^**b**^	**C**^**c**^	**mCI**^**d**^	**FR**^**e**^	**SU**^**f**^
**ACANTHACEAE**									
*Adhatoda vasica* Nees	Bhekker	L	1 kg fresh leaves each of *Adhatoda vasica* and *Rhazya stricta* are soaked in water over night and liquid is orally administered for 3–4 days.	Stomach disorder, fever, dehydration	B, C	45	1.975	-	+
CIITH-2									
			1 kg leaves are mixed with grass or husk and fed to animal for 2–3 days.	Dysentery	B, C, G, Sh				
			½ kg each of leaves of *Adhatoda vasica* and bulbs of *Allium cepa* are crushed and paste is fed to animal for 3–4 days.	Indigestion	B, C, G, Sh, H				
			½ kg fresh leaves are crushed along with table salt and resulting paste is fed to animal for 5–6 days.	Diarrhea, dysentery, gas trouble	A, B, C, G, Sh, Cam				
**AMARANTHACEAE**									
*Aerva javanica* (Burm. f.) Juss.	Chittibui	Wp	1 kg roots are boiled in water and decoction is given orally twice a day for 7–8 days.	Skin infection	B, C, G, Sh	6	0.276	+	-
CIITH-4									
			Whole plant boiled in water and decoction is orally administered at night for 2–3 days.	Intestinal worms	A, B, C, H, M				
*Amaranthus viridis* L.	Chulai	Wp	1 kg fresh plant is crushed and mixed in wheat husk; paste is fed to animal twice a day for 10–15 days.	Against weakness	A, C, Cam, G, M	3	0.648	-	+
CIITH-10									
*Chenopodium album* L.	Bathu	L	Leaves are boiled in mustard oil, and then crushed and topically applied to wounds.	Wound healing	B, C, G, H, Sh	3	0.014	-	+
CIITH-25									
**AMARYLLIDACEAE**									
*Allium cepa* L.	Piaz	B	Bulbs are crushed with sugar. The resulting paste is mixed with milk and orally administered at night and early morning up to 1 week.	Galactagogue	B, C	35	1.839	-	+
CIITH-7									
			½ kg of each leaves of *Adhatoda vasica* and bulbs of *Allium cepa* are crushed and this paste is orally administered for 3–4 days.	Indigestion	B, C, G, H, Cam, Sh,				
			½ kg bulbs are crushed along with salt and mixed in flour. This paste is administered orally for 4–5 days.	Stomach disorder, fever	B, C, G,				
*Allium jacquemontii* Kunth	Jangli- Thoom	B	100 g fresh bulbs are ground and mixed with wheat flour. This paste is applied topically for 10–15 days.	Unequal mammary glands	C, G	2	0.131	+	-
CIITH-8									
**ANACARDIACEAE**									
*Mangifera indica* L.	Aam	F	Pickled fruit is fed to animal for 4–5 days.	Mouth infection	A, B, C, G	3	0.011	-	-
CIITH-51									
**APIACEAE**									
*Anethum sowa *Roxb. ex Fleming	Soay	S	100 g seeds are mixed in wheat flour and orally administered for up to 15–20 days.	Galactagogue	B, C	5	1.028	+	-
CIITH-11									
			100 g each of *Anethum sowa, Trachyspermum ammi* and *Foeniculum vulgare* are ground together and paste is orally administered for 2–3 days.	Abdominal pain, swelling	A, C, Cam, G				
*Foeniculum vulgare* Mill.	Sounf	Ap	200 g of aerial parts are boiled in water. The decoction is mixed with *Trachyspermum ammi, Camelia sinensis,* brown sugar and ghee. This paste is fed to the animal for 5–6 days.	Indigestion	A, B, C, Cam, H, M	22	1.793	-	+
CIITH-46									
			200 g each of *Foeniculum vulgare* aerial parts and *Punica granatum* rind are ground together. The resulting powder is orally administered for 4–5 days.	Diarrhea	B, C, G, H Sh				
*Trachyspermum ammi* (L.) Sprague	Ajwain	S	¼ kg each of *Trachyspermum ammi* seeds*, Anethum sowa*, *Allium cepa* and *Foeniculum vulgare* are mixed in flour; paste is orally administered to animals for 10–15 days.	Appetite stimulant, galactagogue	A, B, B, C, Cam, G, H, M, Sh	39	2.317	-	-
CIITH-79									
**APOCYNACEAE**									
*Calotropis procera* (Aiton) W.T. Aiton	Ak	L	Fresh leaves and black salt are fed to animals for 1–2 days.	Mouth and eye watering	C, Cam, G	15	0.125	-	+
CIITH-19									
		St	The stem is forcefully administered orally.	Colic, indigestion	A, C, H, M				
		Twg	Poultice prepared from young twigs is topically applied.	Pain, inflammation	C, Cam, H, M				
*Carissa opaca* Stapf ex Haines	Granda	L, S	½ kg fresh leaves and ripened seeds are ground and the resulting powder is mixed in water and orally administered to animals for 2–3 days.	Throat infection	G, Sh	3	0.013	+	+
CIITH-23									
		R	100 g dried roots are ground into a powder and sprinkled onto wounds for 2–3 days.	Infected sores, wound healing	B, C, G, Sh				
*Periploca aphylla* Decne.	Kathi	Ltx	Latex is topically applied for 4–5 days.	Skin infection	A, C, Cam, D, H, Sh	5	0.213	+	+
CIITH-57									
*Rhazya stricta* Decne.	Veran	L	Fresh leaves are soaked in water and the resulting liquid is orally administered to animals for 8–10 days.	Skin infection and blood purification	B, C, G, H, Sh	7	0.193	+	-
CIITH-65									
			A decoction of fresh leaves is orally administered for 2–3 days.	Abdominal pain	A, B, C, G, M				
**ARACEAE**									
*Arisaema flavum* (Forssk.) Schott	Adbais	S	8-10 ripened seeds are orally administered.	New Castle disease	P	2	0.103	-	+
CIITH-12									
**ARALIACEAE**									
*Hedera nepalensis* K. Koch	Hurr Bumbal	L	200 g fresh leaves are crushed and soaked in water. The resulting liquid is instilled in the nose twice a day for 1–2 days.	To remove leeches	B, C	4	0.131	+	+
CIITH-48									
**ASTERACEAE**									
*Erigeron* sp.	Taku Booti	Wp	500 g of fresh plant material is crushed and mixed with flour; the paste is orally administered for up to a week.	Fever, stomach collapse	B, C, D, G	14	0.031	+	-
CIITH-40									
*Launaea procumbens* (Roxb.) Ramayya & Rajagopal	Doodh Pathar	L	Paste of fresh leaves is topically applied for 3–4 days.	External worms (skin infection)	A, B, C, D, M	2	0.314	-	+
CIITH-49									
*Saussurea heteromalla *(D. Don) Hand.-Mazz.	Kali Zeri	Wp	50 g seeds are wrapped in paper and fed to animals along with ghee for up to 1 week.	Edema	B, C, H	11	0.874	-	-
CIITH-72									
			200 g dried plant material is ground and the resulting powder is mixed with kneaded flour; paste is orally administered for 8–10 days.	Blood purification	B, C, G, Sh				
*Senecio chrysanthemoides* DC.	Chitta Hola	Wp	Root decoction is orally administered for 5–6 days.	Arthritis	A, C Cam, G, H	4	1.153	-	-
CIITH-73									
			Paste of fresh plant is topically applied.	Sore joints	A, C Cam, G, H				
*Tagetes minuta *L.	Saat Barga	L	Fresh leaves are soaked in water and the resulting liquid is instilled into the ear for 2–3 days.	Earache	A, C, D, G, M	2	0.675	+	+
CIITH-77									
**BERBERIDACEAE**									
*Berberislycium *Royle	Sumbol	Rt	A decoction of ¼ kg bark is prepared and orally administered for 10–12 days.	Bone fracture	A, B, C, G, H, M, Sh,	25	1.753	-	+
CIITH-14									
			100 g bark is ground and powder is sprinkled on wounds up to a week.	Wound healing	A, B, C, G, H, M				
**BORAGINACEAE**									
*Cordia obliqua *Willd.	Lasoora	F	2-3 fruits are mixed in fodder and fed for 4–5 days.	Throat infection, common cold	C, G, Sh	8	0.561	+	-
CIITH-32									
		S	Decoction of seeds is administered orally for 10–15 days.	Stomach ulcer	C, G, Sh				
*Trichodesma indicum *(L.) Lehm. CIITH-81	Hadusi	Wp	Paste of fresh plant is administered orally for a week.	Stomach disorder, intestinal worms	B, C, G, Sh	22	1.769	-	+
**BRASSICACEAE**									
*Brassica campestris* L.	Sarain	S	200 g seeds are ground with 50 g of sulfur and mixed with mustard oil. This paste is topically applied for a week.	Skin infection	A, B, C, Cam, D, H	34	1.359	-	+
CIITH-17									
			100 g seeds are ground, and then powder is mixed with eggs and orally administered for 2–3 days.	Stomach disorder/ infection	B				
		O	2-3 peppers are soaked in mustard oil for a few days and cooked along with bread, which is fed to animals for 8–10 days.	Eye disease (cornea opacity)	B, C				
*Eruca sativa* Mill.	Tara Mira	S	200 ml seed oil is mixed with 200 g of sugar orally administered for 4–5 days.	Dysentery	B, C, G, H	12	1.176	+	-
CIITH-41									
**CANNABACEAE**									
*Cannabis sativa* L.	Bhang	L	100 g of fresh leaves are crushed and paste is applied topically.	Leeches, lice	B, C, G, Sh	5	1.348	-	+
CIITH-21									
			½ kg dried leaves are ground and the resulting powder is mixed with kneaded flour; the paste is fed is to animals twice a day for a week.	Appetite stimulant; abdominal swelling	B, C, D, G, H, M				
		L,Fb,S	1 kg of dried leaves, floral buds and seeds are made into a powder and mixed with wheat flour, salt and water. This paste orally administered for 10–15 days.	Indigestion	B, C, D, G, H, M				
**CAPPARACEAE**									
*Capparis decidua* (Forssk.) Edgew.	Kirir	Ap	½ kg of fresh aerial parts is boiled in water; 1 glass of resulting decoction is orally administered for 5–6 days.	Stomach gripe, indigestion	C, Cam, H, M	4	0.011	+	-
CIITH-22									
**CONVOLVULACEAE**									
*Convolvulus arvensis* L.	Leli	Wp	1 kg fresh plant is crushed along with sugar and water; this juice is given orally for 3–4 days.	Constipation	B, C, G, Sh	9	0.419	+	+
CIITH-31									
*Cuscuta reflexa* Roxb.	NilaTahri	Wp	½ kg plant material is crushed and mixed with flour; paste is applied topically for 8–10 days.	Galactagogue	B, C, G, Sh	5	0.151	-	+
CIITH-35									
			Paste of fresh plant is fed to goats and sheep for 4–5 days.	Indigestion	G, Sh				
**CRASSULACEAE**									
*Bryophyllum pinnatum* (Lam.) Oken	Zakham Hayat	L	Fresh leaves are wormed in mustard oil and bandage on topically on wounds.	Bleeding wounds	C, G, H, M	9	0.086	-	-
CIITH-18									
**CUCURBITACEAE**									
*Citrullus colocynthis* (L.) Schrad.	Tumba	F	Juice is extracted by heating the fruit of *Citrullus colocynth is* and *Calotropis procera* in a mud pot on a garbage fire for 2–3 weeks; it is orally administered for up to a week.	Indigestion, gas trouble, abdominal worms	B, C, Cam, D, G, H, M, Sh	17	0.514	+	-
CIITH-27									
			100 g fruit is crushed and mixed in *Aloe vera* pulp. This paste is orally administered for 2–3 days.	Constipation	B, C, Cam, D, Sh				
*Citrullus vulgaris* Schrad.	Rainda	F	1 kg fresh fruit coat is ground with salt and orally administered for 10–12 days.	Appetite stimulant, galactagogue	A, C, Cam, G	13	0.417	+	-
CIITH-28									
*Cucumis melo* L.	Chibber	L, F	Paste from fresh leaves and fruits are fed to animals for up to 1 week.	Indigestion	B, C, G, Sh	5	0.463	-	-
CIITH-33									
**EUPHORBIACEAE**									
*Euphorbia wallichii* Hook. f.	Hervi	Ltx	50 ml stem latex is topically applied for 2–3 days.	Rashes, wound healing	A, C, Cam, D, G, M	3	0.041	+	+
CIITH-43									
*Mallotus philippensis* (Lam.) Müll. Arg.	Kamila	F	¼ kg dried fruits are ground and the resulting powder is fed to animals along with wheat flour for 2–3 days.	Intestinal worms	C, G, Sh	21	1.103	-	+
CIITH-50									
*Ricinus communis *L.	Hernoli	S	½ cup of seed oil is orally administered for up to a week.	Constipation	B, C, Cam, G, H, Sh	8	0.052	-	+
CIITH-66									
**FABACEAE**									
*Acacia nilotica* (L.) Willd. ex Delile	Kiker	Bk	Decoction of ½ kg bark is orally administered twice a day for 5–6 days.	Stomach disorder	B, C, H	10	0.686	-	+
CIITH-1									
*Cassia fistula* L.	Kinjal	S	4-6 seeds are mixed with chicken feed and fed to hens.	Newcastle disease	P	3	0.413	-	+
CIITH-24									
*Cicer arietinum* L.	Kalay	S	200 g seeds are ground and resulting powder is mixed with yogurt; this paste is orally administered for 10–15 days.	Piles	C	2	0.139	-	-
CIITH-26									
*Dalbergia sissoo* Roxb. ex DC.	Tahli	L	½ kg fresh leaves *of Dalbergia sissoo* and 200 g linseeds are boiled in water. This decoction is administered orally for 8–10 days.	Constipation	C, G	3	0.117	-	+
CIITH-37									
*Phyllodium pulchellum *(L.) Desv.	Ladan	Rt	Root decoction is administrated orally for 10–15 days.	Fever, weakness	A, B, C, H, M	12	0.075	+	+
CIITH-39									
*Trigonella foenum-graecum* L.	Mathray	S	200 g seeds are ground and the resulting powder is used orally after washing urethra with a sugar and potash alum (potassium alum) mixture for 4–5 days.	Urethra prolapse	B, C	13	1.541	-	-
CIITH-82									
			50 g seeds are mixed with fodder and fed to animal for 3–4 days.	Diarrhea	B, C				
**LAMIACEAE**									
*Ajuga bracteosa* Wall. ex Benth.	Ratti Booti	Wp	125 g shade-dried plant is ground and resulting powder is mixed with flour and orally administered for 2–3 days.	Abdominal pain	B, C, G	3	0.433	-	+
CIITH-6									
**LYTHRACEAE**									
*Punica granatum* L.	Druni	Fr	¼ kg dried rind is ground and the resulting powder is fed to animals along with flour up to 1 week.	Dysentery	C, G, Sh	9	1.769	-	+
CIITH-63									
**MELIACEAE**									
*Melia azedarach* L.	Dhrek	L	200 g fresh leaves are crushed along with sugar and water; the mixture is administered orally to animals for 2–3 days.	Foot, mouth infection	G, Sh	29	2.101	-	+
CIITH-52									
		Tw	100 g fresh twigs are crushed and soaked in water; the resulting liquid is given orally for 2–3 days.	Skin infections	B, C, G, H				
		Fr	200 g fruit rinds are soaked in water and the resulting juice is given orally for 4–5 days.	Rashes	B, C, G, H				
		L,Tw,F	200 g of fresh leaves, twigs and fruits are crushed. This paste is fed to animals up to a week.	Gas trouble, indigestion	B, C, G, H, Sh				
**MORACEAE**									
*Ficus palmata* Forssk.	Phagwar	L, F	½ kg dried leaves and fruits are ground and the resulting powder is administered orally with water for 5–6 days.	Indigestion	C,G	2	0.316	-	+
CIITH-45									
**MYRTACEAE**									
*Eucalyptus camaldulensis* Dehnh.	Safada	L	Fresh leaves are fed to animals for 4–5 days.	Common cold	B, C, G	4	0.010	+	-
CIITH-42									
*Syzygium cumini* (L.) Skeels	Jaman	L	Fresh leaves are fed to animals.	Diarrhea	B, C, G	3	0.010	+	-
CIITH-76									
**NITRARIACEAE**									
*Peganum harmala *L.	Hremal	L,Br	Smoke of leaves and branches is used for 4–5 days.	Mastitis	B, C, H	17	1.736	-	-
CIITH-56									
		L	½ kg fresh leaves ground with salt; paste is orally administered for 5–6 days.	Gastric problems	B, C, Cam, H, M				
		S	200 g dried seeds are burnt and mixed in mustard oil. This infusion is applied topically 2–3 days.	Ticks and mites	B, G, Sh				
**OLEACEAE**									
*Olea ferruginea* Royle.	Kahu	F	Extract of fruits is given orally for 5–6 days.	Indigestion	C, G	3	0.017	+	+
CIITH-54									
**PAPAVERACEAE**									
*Fumaria indica* (Hausskn.) Pugsley	Papra	Wp	Fresh plant material is fed to animals for 2–3 days.	Diarrhea	C, G	6	1.511	-	+
CIITH-47									
**PLANTAGINACEAE**									
*Plantago lanceolata* L.	Batti	L	Leaf paste is topically applied.	Neck rashes	C, H	2	0.173	+	+
CIITH-58									
*Plantago major* L.	ChimchipAtra	Wp	200 g dried plant is ground and the resulting powder is sprinkled on infected hooves for 6–7 days.	Infected hooves	C, Cam, G, H, M	3	0.185	-	+
CIITH-59									
**POACEAE**									
*Cynodon dactylon* (L.) Pers.	Khabul	Wp	100 g of fresh plant material is ground and the paste is topically applied for 2–3 days.	Wound healing	A, B, C, D, H, M	7	0.351	-	+
CIITH-36									
*Oryza sativa* L.	Chawal	S	1 kg rice is boiled in water along with yoghurt and *Eruca sativa* oil; paste is fed to animals for 15–20 days.	Weakness, lung infection	B, C, Cam, H, M	6	0.075	-	-
CIITH-55									
*Saccharum bengalense* Retz.	Kana	Rt	½ kg roots are boiled along with ½ kg *Solanum surattense;* decoction is orally administered for 8–10 days.	Intestinal worms, appetite stimulant	C, G	3	0.010	+	+
CIITH-71									
*Sorghum halepense* (L.) Pers.	Baru	Rt	Fresh roots are crushed and soaked in water; the resulting liquid is orally administered for 2–3 days.	Indigestion	D	3	0.011	-	-
CIITH-75									
*Triticum aestivum* L.	Kank	S	200 g seed porridge is given orally to animals for a week.	Galactagogue, dysentery	B, C, G	27	1.753	-	+
CIITH-83									
			Hot bread is fed to cattle for a week.	Mouth sores	C, G, Sh				
			¼ kg seeds are ground with brown sugar and the resulting paste is fed to animal for 8–10 days.	Galactagogue	B, C, G				
*Zea mays* L.	Maki	Stg	A decoction of the female inflorescence isorally administered for 4–5 days.	Urinary inflammation	B, C, G	15	0.015	-	+
CIITH-88									
**POLYGONACEAE**									
*Polygonum amplexicaule* D. Don.	Mosloon	Rt	Root decoction is orally administered to animals for 1 week.	Dehydration, fever	B, C, G, Sh	2	0.138	+	+
CIITH-60									
		L	Fresh leaves are fed to animals for 3–4 days.	Indigestion	B, C, G				
*Polygonum plebeium* R. Br.	Sarwar Booti	Wp	Paste of the fresh plant is applied topically for 2–3 days.	Scorpion bite	B, C, D, H	3	0.109	+	-
CIITH-61									
*Rumex dentatus* L.	Jngli	Rt	Root decoction is orally administered for up to a week.	Foot, mouth infection	C, G, Cam, H, M	4	0.014	+	+
CIITH-68	Palak								
*Rumex hastatus* D. Don	Khitiml	Ap	Arial parts are used as a brush for a week.	Scabies	B, C	3	0.915	-	+
CIITH-69									
		Rt	1 kg of each of *Rumex hastatus* roots and *Quercus incana* bark are boiled; decoction is mixed in sugar and flour. This sweet meal is fed to animals for 8–10 days.	Cough, fever, weakness	B, C, G				
*Rumex nepalensis* Spreng.	Hoola	L	Extract of fresh leaves is topically applied to infected parts for 4–5 days.	Antiseptic and anti-inflammatory	C, G, M, Sh	2	0.013	-	+
CIITH-70									
			Leaf paste is applied topically for 2–3 days.	Hemostatic	A, B, C, D				
		Rt	½ kg fresh roots are crushed with salt and the resulting paste is administered orally for 5–6 days.	Diarrhea, dysentery, and intestinal worms	G, Sh				
**PRIMULACEAE**									
*Myrsine africana* L.	Khukan	L	Fresh leaves are fed to animals for up to a week.	Indigestion, worms	G,Sh	17	0.735	+	+
CIITH-53									
**PTDERIDACEAE**									
*Adiantum incisum* Forssk.	Sarhaaj	L	Paste of crushed leaves is made by mixing with wheat flour and orally administered for 2–3 days.	Abdominal pain	B, C	5	0.336	+	+
CIITH-3									
**RANUNCULACEAE**									
*Clematis grata* Wall.	Dhand	L	Paste of fresh leaves is applied topically on infection sites.	To kill external worms in wounds	A, C, Cam, D, M	17	0.432	+	+
CIITH-30									
**RHAMNACEAE**									
*Ziziphus nummularia *(Burm. f.) Wight & Arn.	Beri	L	100 g leaves are boiled and decoction is orally administered for 1–2 days.	To discharge placenta following birth	B, C	7	0.018	-	+
CIITH-89									
**ROSACEAE**									
*Prunus persica* (L.) Batsch	Aru	L	Juice of fresh leaves is applied topically for 4–5 days.	To kill germs/worms	A, B, C, D, H, M	21	1.783	-	+
CIITH-62									
			Leaf decoction is orally administered for 2–3 days.	Dysentery	G, Sh				
*Pyrus pashia* Buch.-Ham. ex D. Don	Batangi	F	Powder of dried fruits is given orally for 5–6 days.	Dysentery, diarrhea	B, C	4	0.241	-	+
CIITH-64									
*Rosa cymosa *Tratt.	Ghulab	Fl	100 g flowers are soaked in a sugar solution and mixed with milk; mixture is orally administered for 2–3 days at 10–20 days post-delivery.	To clean uterus	B, C	3	0.101	-	+
CIITH-67									
**RUTACEAE**									
*Citrus limon* (L.) Osbeck	Nimbu	F	Fruit juice is mixed with sugar and this paste is fed to animals and applied topically (to the mammary glands) for 10–15 days.	Mastitis	B, C, G	8	0.131	-	-
CIITH-29									
*Zanthoxylum armatum* var. *armatum*	Timer	L	100 g leaves are crushed and mixed with kneaded flour; the resulting paste fed to animals for 3–4 days.	Vomiting, indigestion	B, C, G, Sh	38	2.101	-	+
CIITH-87									
		S,F	50 g seeds/fruit are orally administered with flour.	Indigestion, mouth infection	B, C, G, Sh				
**SAPINDACEAE**									
*Aesculus indica* (Wall. ex Cambess.) Hook.	Bankhor	F	¼ kg fruits are ground and the powder mixed with husk and fed to animals for 5–6 days.	Cough, fever, abdominal pain	A, B, C, Cam, G, H, M	18	1.576	-	+
CIITH-5									
			Fruit juice is applied topically up to a week.	External wounds	B, C				
**SAXIFRAGACEAE**									
*Bergenia ciliata* (Haw.) Sternb.	Batpia	Rh	Fresh leaves are slightly crushed and applied as bandages on bleeding wounds.	Hemostatic (for bleeding wounds)	B, C	15	1.341	-	+
CIITH-15									
			Dried rhizome powder is sprinkled onto wounds for 8–10 days.	Wound healing	A, B, C, G, D, M				
*Bergenia stracheyi* (Hook. f. & Thomson) Engl.	Batpia	Rh	Dry root powder is sprinkled onto wounds for 8–10 days.	Wound healing	A, B, C, G, D, M	12	0.785	+	-
CIITH-16									
**SCROPHULARIACEAE**									
*Verbascum thapsu s *L.	Gidhar Tumaku	Wp	100 g fresh plant is crushed and paste is fed to animal for a week.	Diarrhea	C, D	2	1.135	-	+
CIITH-85									
**SOLANACEAE**									
*Datura innoxia* Mill.	Datura	L	Leaf extract is applied topically for 1–2 days.	Anti-lice	B, C	9	0.543	-	+
CIITH-38									
*Solanum surattense* Burm. f.	Mhokri	Wp	Fresh fruit paste is applied topically for 2–3 days.	Wound healing	B, C, G, D, H, M	18	0.984	-	+
CIITH-74									
			½ kg fresh plant is cooked with salt, peppers and yoghurt. This paste is orally administered for 10–15 days.	Tonic	B, C, G, H, Sh				
			200 g fresh plant is boiled along with black pepper and salt; decoction is given orally for 8–10 days.	Fever, indigestion, cough	C, G				
*Withania somnifera* (L.) Dunal	Aksan	Rt	200 g fresh roots are crushed and paste is applied topically up to a week.	Mastitis	B, C, G	8	0.736	-	+
CIITH-86									
**TAMARICACEAE**									
*Tamarix aphylla* (L.) H. Karst.	Rokh	L	An infusion of the dried, burnt leaves is applied topically onto the skin, nose and ear in livestock.	To kill external worms in wounds	A, C, D, H	5	0.011	+	-
CIITH-78									
**THEACEAE**									
*Camellia sinensis* (L.) Kuntze	Chay	L	½ kg fresh leaves are boiled in water along with sugar; 1 glass of this decoction is given orally for 5–6 days.	Fever	C, G	4	0.101	+	+
CIITH-20									
**VITACEAE**									
*Vitis vinifera* L.	Dakh	L, Wd	Ash prepared from wood and leaves of is orally administered with milk for 8–10 days.	Hemoglobinuria	C, G	3	0.191	+	+
CIITH-84									
**XANTHORRHOEACEAE**									
*Aloe vera* (L.) Burm. f.	Kwar Gandal	L	½ kg of leaf pulp, salt and *Trachyspermum ammi* are mixed and paste is administered orally for up to 1 week.	Digestive problems	C, G, H	8	0.175	-	+
CIITH-9									
*Asphodelus tenuifolius* Cav.	Bokhat	L	Fresh leaves are crushed and fed to horses for 8–10 days.	Constipation	A, C, Cam, H	3	0.037	+	-
CIITH-13									
**ZINGIBERACEAE**									
*Curcuma longa* L.	Haldi	Rt	Powder from dried roots is applied and wrapped or sprinkled onto wounds for 2–3 days.	Wound healing	A, C, Cam, H	4	2.181	-	-
CIITH-34									
**ZYGOPHYLACEAE**									
*Fagonia indica* Burm. f.	Dhamian	Wp	Fresh leaves are fed to animals for 8–10 days.	Appetite stimulant, indigestion	B, C, Cam, G, H	2	0.015	+	-
CIITH-44									
*Tribulus terrestris* L.	Bhakra	L	200 g of each of *Tribulus teristri* dried leaves, *Curcuma domestica* and *Foeniculum vulgare* are ground together and the resulting powder is orally administered to cattle for 10–15 days.	Appetizer, joint pain	B, C, Cam, G, H	7	0.472	+	+
CIITH-89									
			Paste of fresh plant is orally administered for up to a week.	Gastric problems	B, C				

^a^Part(s) of the plant used: Ap: Aerial parts; Bk: Bark; Br: Branches; B: Bulb; L: Leaves; Fb: Flower buds; F: Fruit; Fl: Flower; Fr: Fruit rind; Rh: Rhizome; S: Seed; Rt: Root; O: Oil; St: Stem; Stg: Stigma; Twg: Twigs; Ltx: Latex; Wd: Wood; Wp: Whole plant.

^b^AT: Animals treated. A: ass; B: buffalo; C: cow; Cam: camel; D: dog; Dk: donkey; G: goat; H: horse; M: mule; P: poultry, Sh: sheep; Sw: swine.

^c^C: Number of citations.

^d^mCI: mean cultural importance index value.

^e^FR: First report of this Ethnoveterinary practice in the Himalayan region. +: yes; -: no.

^f^SU: Similar use/application in local folk medical practice for humans.+: yes; -: no.

### Most versatile and used veterinary plants

Of the 89 recorded plant species, frequently applied plant species against veterinary ailments included: *Adhatoda vasica, Calotropis procera, Melia azedarach, Rumex nepalensis* (6 diseases); *Cannabis sativa* (5); *Aesculus indica, Allium cepa, Citrullus colocynthis* and *Rumex hastatus* (4). *Adhatoda vasica* was the most cited species (43%), followed by *Trachyspermum ammi* (37%), *Zanthoxylumarmatum* var. *armatum* (36%), *Allium cepa* (33%), and *Brassica campestris* (32%). Based on the diversity of conditions treated by plants in each family, the Polygonaceae family was found to have the broadest application with 8 recipes for the treatment of 17 veterinary conditions (8/17), followed by Poaceae (7/10), Asteraceae and Fabaceae (7/7), and Apiaceae and Cucurbitaceae (5/7).

### Plant parts used, their preparations and applications

Among the plant parts included in veterinary applications, leaves were most commonly used (26%), followed by seeds (13%), whole plant (13%), and fruits (11%). The methods of preparation of the therapeutic materials sometimes varied from individual to individual (e.g., the same plant material for the same ailment could be prepared in different ways, depending upon the preferences of different healers). A list of 126 ethnoveterinary remedy preparations is presented in Table 1. The large majority of recipes being were prepared from single plants (70%) rather than mixtures. In most cases, water was the solvent employed in preparation of the remedy. Besides plants and water, some other materials were also commonly incorporated in the preparations: salt, sugar, milk, oil, eggs and ghee. The most common therapeutic formulations fall into eleven main categories, the most popular of which were pastes (35%), fresh plant parts (15%), decoctions (14%), and powders (13%).

### Ethnoveterinary plant uses

Study participants identified more than 50 veterinary ailments that could be grouped into seven general categories: gastrointestinal disorders (these were treated by 47 formulations); skin infections (30), parasites, fever and respiratory diseases (10), reproductive disorders (9), lactation (4), and musculoskeletal system disorders (3). More than 40 taxa were documented for their application to treat more than two veterinary conditions.

By comparing the present data with all of the available ethnoveterinary literature concerning the surrounding geographic areas, it appears that nearly half of the quoted plants have never been described before as useful in folk veterinary practices. The other half has already been reported in the literature, but in some cases, for different ethnoveterinary purposes. In this section, we explore some reports on other ethnoveterinary applications of these species in the literature. This discussion is organized by plant family.

### Acanthaceae

Regarding *Adhatoda vasica*, the leaves are used to treat stomach pains, fever, dehydration, diarrhea, dysentery, indigestion and gas troubles. The leaf paste of this plant has been reported for uses in the treatment of hoof rot in the literature [46]. Interestingly, aqueous extracts from the leaves have shown significant activity against *Bacillus* bacteria [47,48].

### Amaranthaceae

Paste prepared from whole plant of *Amaranthus viridis* is used here against weakness in cattle. The leaves of the same plant were reported as emollient in amenorrhea, scorpion sting and snake bite in a study conducted in Islamabad, Pakistan [49].

### Amaryllidaceae

The crushed bulbs of *Allium cepa* are administered to treat indigestion, stomach gripe, fever and for lactation in the study area, whereas in Italy, they are used to prevent pestilence [50]. The leaves, flowers and bulb extracts of *A. cepa* have demonstrated activity against pathogens such as *Staphylococcus aureus, Salmonella typhi*, *Candida albicans,* and nematodes [48,51].

### Anacardiaceae

The fruit pickle of *Mangifera indica* is used for mouth infections. Others have reported that the leaves of same plant are fed to livestock to treat retained fetal membrane [20]. Chloroform, ethanolic, water and petroleum ether extracts of *M. indica* were found to have anti-bacterial, anti-viral anti-fungal activities, as well as anti-inflammatory properties [52,53].

### Apiaceae

Aerial parts of *Foeniculum vulgare* were used to treat indigestion and diarrhea. Flowers and fruit of the same species have been reported as galactagogues and ruminative [50]. Seeds of *Trachyspermum ammi* are given to cattle as appetite stimulant and to increase milk production. In the Sargodha district of Pakistan, seed powder and decoctions of the same plant were reported for treatments against genital prolapse and to treat retained fetal membrane [20]. Alcoholic and aqueous extracts of this plant species have shown antibacterial activity [54].

### Apocynaceae

Leaves, stems and twigs of *Calotropis procera* are applied to cure mouth and eye watering, colic, indigestion, pain and inflammation. Other reports regarding use of this plant include crushed leaves for the relief of flatulence, latex to increase lactation and bark decoction for hoof rot [46]. The leaves and seeds are also reported to be useful for silent estrus and delayed puberty [20]. Alcoholic and aqueous extracts of *C. procera* have shown antibacterial activities [54]. Powder prepared from the roots and leaves of *Carissa opaca* is given to cattle to treat infected or sore throats and to heal wounds. In Uttar Pradesh, India, aerial parts of *C. opaca* were reported to be administered orally to kill pest in cattle [46].

### Araliaceae

An aqueous extract of *Hedera nepalensis* is applied to remove leech in cattle. In Italy, the use of fresh leaves and plant decoctions for abortive and anti-inflammatory purposes have been reported [50].

### Asteraceae

The seeds and paste made from the whole plant of *Saussurea heteromalla* are used to treat edema and to purify the blood. In Islamabad, the seeds were reported as carminatives and used also in tonics for horses and camels [49]. In the present study, we found that decoctions and pastes of *Senecio chrysanthemoides* are used for the treatment of sore joints and arthritis, whereas other work has reported the use of roots and leaves for treating blackleg disease and Evil-eye [55].

### Boraginaceae

The leaf paste of *Trichodesma indicum *is used to treat stomach disorders and intestinal worms in cattle in the study area, whereas others have reported the use of this paste in the treatment of mastitis and for uterine prolapse [46].

### Brassicaceae

*Brassica campestris* seed oil is used for skin, eye and stomach infections. Other studies in Pakistan and India [20,46] have reported the use of this oil in topical applications for sores and the treatment of genital prolapses. *Eruca sativa* seed oil is used to treat dysentery in the study area. *E. sativa* seed powder has been reported for diarrhea in other work [46].

### Cannabaceae

Paste from the leaves, seeds and floral buds of *Cannabis sativa* are applied as an appetite stimulant, anti-leech, anti-lice, and for abdominal swelling and indigestion. Other studies have reported the use of decoctions and infusions for measles and East coast fever [56] and leaves for genital prolapse [20].

### Convolvulaceae

Paste prepared from *Cuscuta reflexa* is fed to cattle for treatment of swelling (rumination problems), indigestion and short mammary glands. Other studies have documented its use as a galactagogue food (after being fried) [57].

### Euphorbiaceae

Seed oil of *Ricinus communis* is administered to treat constipation. Other studies have documented the use of *R. communis* for intestinal obstruction, hoof problems, digestive problems, wounds, abscesses, to expel retained placenta and for silent estrus/delayed puberty in cattle [20,46,58]. The stem/leaf hexane extract of *R. communis* was suggested to be active against *Escherichia coli, Enterococcus faecalis, Pseudomonas aeruginosa* and *Staphylococcus aureus*[59].

### Fabaceae

*Acacia nilotica* bark decoctions are used for the treatment of stomach pains in livestock. The bark of this same plant has been reported to be used in the case of hoof rot and genital prolapse in cattle [20,46]. The seeds of *Trigonella foenum-graecum* are reported to treat diarrhea here, whereas in other areas of Pakistan they are used for treatment of genital prolapse, silent estrus and delayed puberty [20].

### Lythraceae

The fruit rind of *Punica granatum* is used to cure dysentery. Other work reports the use of leaf paste for enteritis, bark powder for helminthic infection, flowers as a tonic and the rind as an astringent and to treat diarrhea [60]. Antibacterial studies on the alcoholic and aqueous extracts of this plant have demonstrated activity against *Bacillus subtilis, Escherichia coli, Proteus vulgaris, Salmonella typhimurium, Pseudomonas aeruginosa* and *Staphylococcus aureus*[51].

### Meliaceae

The leaves and fruit of *Melia azedarach* are used against foot, mouth, skin infections, gas trouble and indigestion in the study areas while according to other studies; it is used as a cooling agent and for genital prolapse [20,46].

### Ptderidaceae

*Adiantum incisum* leaf paste is used for abdominal pain in the study area, whereas in Italy, a decoction of the plant is used to expel the placenta following delivery [50].

### Poaceae

Paste prepared from the seeds of *Oryza sativa* is used to treat weakness and respiratory infection. It was reported [20,50] that seeds of the same plant are also used against diarrhea and to treat retained fetal membrane. *Triticum aestivum* seeds are used against dysentery, sore mouth and to increase milk production in livestock. Other studies have reported its use as a ruminative, laxative, for dermatitis, delayed puberty, silent estrus and to treat retained fetal membrane [20,50]. *Zea mays* inflorescences are given to cure urinary inflammation in cattle. *Z. mays* has been reported for applications in wound healing and treating genital prolapse in other studies [20,50].

### Polygonaceae

Local people use the roots and leaves of *Rumex nepalensis* for treating diarrhea, dysentery, intestinal worms, allergies and to stop bleeding in cattle. Crushed roots of this plant have been reported for treatment of blackleg disease (an infectious disease attributed to *Clostridium* spp.) [55].

### Rutaceae

*Citrus limon* juice is used in the treatment of mastitis. Others have reported the use of citrus juice for uterine prolapse in cattle [20].

### Sapindaceae

The powder and juice of *Aesculus indica* fruit and seeds is used against cough, fever, abdominal pain and to heal wounds in animals in the study area. However, in other regions of Pakistan, the seed endocarp is given to horses to relieve stomach pain, colic and swelling [61-63].

### Saxifragaceae

Fresh leaves and powder derived from the rhizomes of *Bergenia ciliata* are topically applied for use in wound healing. Dried and fresh leaves of the same plant have been used to treat diarrhea in animals [64]. Alcoholic and aqueous extracts of *B. ciliata* rhizome has shown antibacterial and antifungal activities [65].

### Scrophulariaceae

The fresh leaf paste of *Verbascum thapsus* is used to treat diarrhea. Others report the use of a leaf ointment for the treatment of rectal prolapse [50].

### Solanaceae

*Solanum surattense* is used for healing wounds, fever, indigestion, cough and as a tonic. Others have reported the use of the leaves for genital prolapse [20]. A leaf extracts of *S. surattense* was found to be active against *Staphylococcus aureus, Salmonella typhi*, *Candida albicans* and nematodes [48,51]. The root paste of *Withania somnifera* is topically applied to treat bovine mastitis in this study area, whereas the crushed roots of this same species are used against an evil spirit (Wan laffa) in animals in Ethiopia [55]. Alcoholic and aqueous extracts of *W. somnifera* have shown antibacterial activity against *Bacillus subtilis, Escherichia coli, Proteus vulgaris, Salmonella typhimurium, Pseudomonas aeruginosa* and *Staphylococcus auerus,* as well diuretic and anti-hypercholesterolemic activities [48,54].

### Theaceae

Decoctions of *Camellia sinensis* leaves are used to cure fever in cattle in this region, while another study in Sargodha district (Pakistan) reported the use of this decoction for treating retained fetal membrane in cows [20]. Fermented tea has been shown to be hypolipidemic and to reduce high blood pressure [49].

### Xanthorrhoeaceae

The leaf pulp of *Aloe vera* is administered orally as ruminative. The pulp of this same species has also been reported for similar use in the treatment of digestive problems [58]. Alcoholic and aqueous extracts of this plant have shown significant activity against *Bacillus subtilis, Escherichia coli, Proteus vulgaris, Salmonella typhimurium, Pseudomonas aeruginosa* and *Staphylococcus aureus*[54,66,67]. Leaves of *Asphodelus tenuifolius* were used to cure weakness in horses in our study, while others have reported that root paste of this plant is applied to wounds in cattle [46].

### Zingiberaceae

Turmeric powder (from *Curcuma longa* rhizomes) is topically applied for wound healing in cattle in the study area, while a study on equine medicines has mentioned that roots of this plant are used for hoof problems and sore joints [58]. Alcoholic and aqueous extracts of *C. longa* have shown antibacterial activity [54]. Chloroform, ethanol, water and petroleum ether extracts of *C. longa* rhizome were also found to be active against bacteria, viruses, and fungi, and have shown anti-inflammatory activities [52,53].

### Cultural importance of the species

The Cultural Importance index (CI) of species is useful for estimating the significance of certain plants to a given culture [68] and takes into account not only the spread of the use (number of informants) for each species, but also its versatility, i.e. the diversity of its uses [68].

Based on medicinal applications, *Trachyspermum ammi* was found to be the most cited species followed by *Curcuma longa, Melia azedarach, Zanthoxylum armatum* var. *armatum, Adhatoda vasica, Allium cepa, Foeniculum vulgare, Prunus persica, Punica granatum, Trichodesma indicum, Berberis lycium, Triticum aestivum* and *Peganum harmala* (Table 1). It is notable that the top ten species of medicinal plants used to treat various livestock conditions were cited in all three major study sites (Haripur, Abbottabad, and Mansehra).

### Cultural importance of the families

With regards to the diversity of species used, Fabaceae and Poaceae were the most important, with 6 species cited. Like the study by Pardo-de-Santayana et al. [30], we also elected to add the sum of CI of species in each family in order to measure the mean cultural importance of the families (mCIf). Unlike the aforementioned study, however, the number of species reported here did not strongly correlate with the number of species (R^2^ = 0.211). This could be explained, perhaps, by the greater diversity of families (with a more limited number of species per family, average of 1.9 species/family) quoted for ethnoveterinary applications. Of the families reported, Apiaceae had the highest mCIf value, despite having only three species in this group (Figure 2).

### Informant consensus

Perceived efficacy of medicinal plants can be assessed by ICF values, with those plants that are supposed to be effective in curing diseases having elevated ICF levels [69]. We identified seven major disease categories and the highest ICF values were recorded for respiratory disorders and fever (0.68), followed by reproductive disorders (0.63), worms and other parasitic diseases (0.63) (Table 2).

**Table 2:** Informant consensus values based on categories of veterinary conditions

**Category**	**Botanical taxa used**	**Plant reports**	**ICF value**
Gastrointestinal disorders (incl. tympany, colics)	47	70	0.33
Skin diseases (incl. wounds and diseases affecting eye, ear, and throat)	30	54	0.45
Diseases related to milk production	4	7	0.50
Rheumatoid disorders and inflammations	3	5	0.50
Worms and other parasitic diseases	14	35	0.62
Reproductive disorders	9	24	0.63
Respiratory disorders (cold, cough) and fever	10	29	0.68

### Comparison with human medicine

A large number of the veterinary plant reports share commonalities with the folk medical practices used in traditional ethnomedicine for humans in surrounding sites (last column in Table 1). This overlap may be a reflection on how folk veterinary remedies may be the diachronic result of a deep observation of the efficacy of certain plants used in animal diseases or at least of intense transfers of local knowledge between the folk veterinary and the ethnomedical domains.

### Bio-conservation concerns

**Figure 3:**
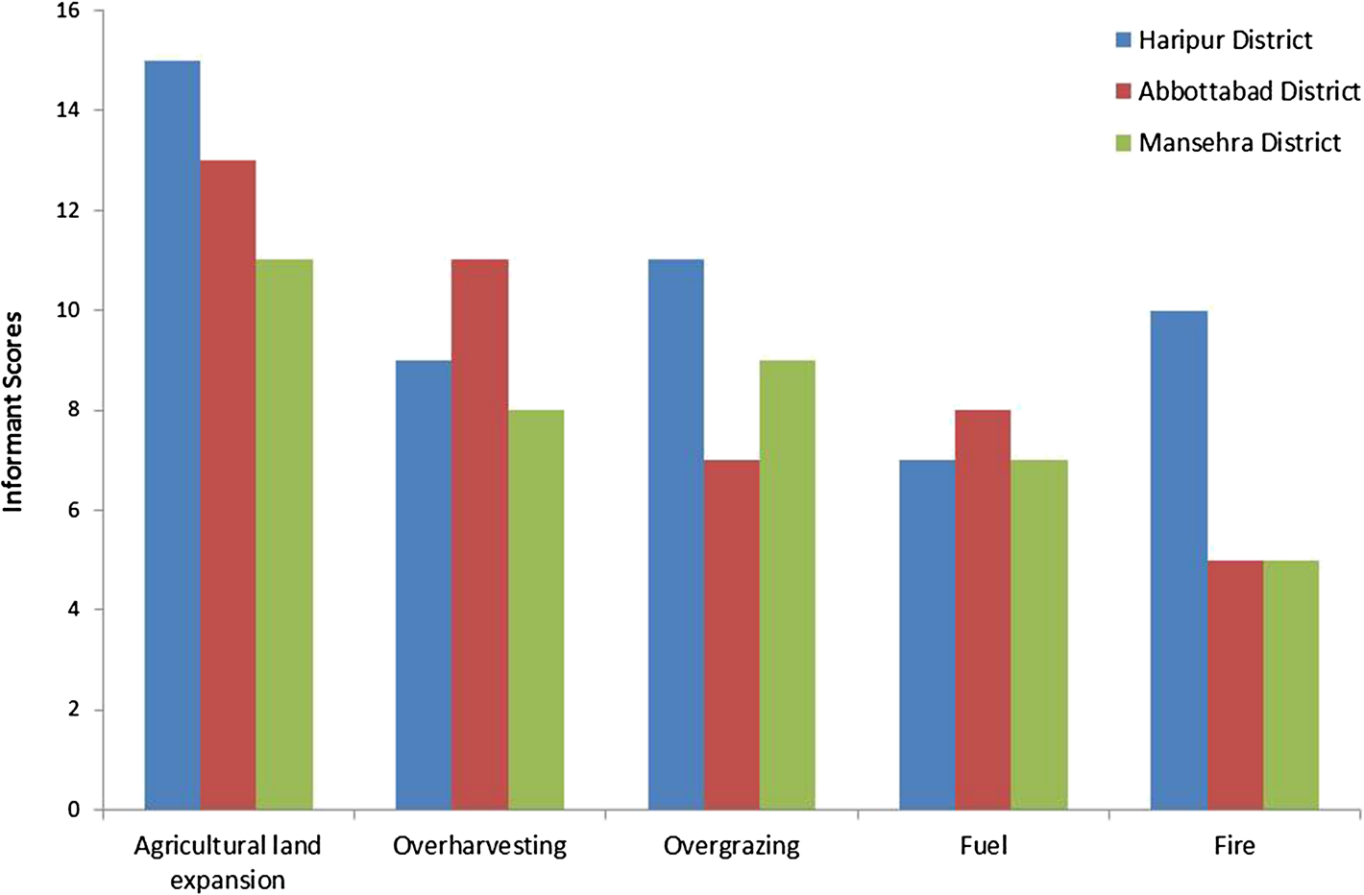
**Factors perceived to be threats to the local medicinal flora.** Ranking based on interviews from the three districts included in this study.

Various human activities may be implicated in placing some of the local medicinal flora under a state of threat within their natural habitat. The perceptions that local people share regarding this phenomenon of threats to local ecological resources – medicinal plants, in particular, was examined based on interviews with 15 key respondents in study districts. We examined these perceived threats using pair-wise ranking [29] of five central factors: agricultural land expansion, over-harvesting, over-grazing, uncontrolled fire setting and fuel wood collection. It was observed that agricultural land expansion was perceived as the dominant threat to medicinal plants used in ethnoveterinary medicine, followed by over-harvesting, over-grazing, fire and fuel wood collection (Figure 3). Current conservation efforts concerning medicinal plants in this region are very limited, and as a result, the majority of them have no protection. This a major issue to be considered in future research and in local rural development initiatives.

## Conclusions

A remarkable heritage of folk veterinary knowledge has been preserved within the framework of local knowledge and practices in the Pakistani communities of the Lesser Himalayas. However, like many other studies in this discipline have found, local knowledge is fragile and susceptible to rapid erosion with the expansion of biomedical paradigms and replacement of traditional resources with modern allopathic medicines. This is increasingly the case in both human and veterinary medicine. Nevertheless, as the majority of the reported species are wild and sometimes rare or under threat, much heed must be taken not to diminish these plant populations.

It is more urgent now than ever to record this rich body of knowledge not only for the purpose of bio-cultural conservation, but also to provide insights to scientists engaged in the search for new herbal veterinary therapies and especially to local stakeholders, who work on fostering endogenous trajectories of community-based rural development projects in mountainous areas. The latter perspective is of crucial importance in the possible implementation of ethnobiological studies in disadvantaged areas, such as the mountain regions of Pakistan [70-73] as it may have a tremendous impact in sustaining and/or revitalizing communal forms of natural resource management [74]. Moreover, e*mic* visions of environmental protection and provision of health and dietary care both for humans and animals may represent the key to environmental and social sustainability of social-ecological systems [75]. The validation and eventual application of this knowledge into concrete, comprehensive and culturally appropriate participatory initiatives aimed at fostering the sustainable use of local natural resources would promote the well-being of both animals and local communities.

## Competing interest

Authors declare that they have no competing interest.

## Authors’ contributions

AMA conducted the ethnobotanical survey and drafted the manuscript; SMK helped in the data compilation; MA supported the field data collection; MAK supervised the project and helped in plant identification; CLQ analyzed the data and reviewed the manuscript; AP critically reviewed the manuscript and wrote the discussion and the conclusions. All authors read and approved the final manuscript.
